# Reviews of Books

**Published:** 1923-11

**Authors:** 


### REVIEWS OF BOOKS.

### MYTH AND RELIGION.

**Folklore in the Old Testament.** (Abridged Edition.) By Sir JAMES GEORGE FRAZER, F.R.S., F.B.A. (Macmillan and Co. 18s. net.)

To a volume of essays published in 1907 in honour of Dr. E. B. Tyler's seventy-fifth birthday, Sir James Frâzer made a contribution on the Folklore of the Old Testament. This study grew afterwards into the three large volumes which appeared in 1918. As was the case with "The Golden Bough," the size of the work tended to some extent to limit its usefulness. Accordingly the author has now issued an abridged edition of both works. Both, however, under his hand have retained the same form and general argument with a reduction chiefly of the footnotes and references.

The subject, indeed, has a special fascination in that the main outlines of the stories are familiar to almost every one of us, and from their associations with the teaching of our childhood have an emotional content which is not possessed by any other subject. The stories of the Covenant of Abraham, of the heirship of Jacob and his struggle with the mysterious adversary at the ford of Jabbok, the witch of Endor, Samson and Delilah, to mention a few only which fill these pages, will be read with a new interest in the light thrown upon them by Sir James Frazer. He has a fine power of description which brings the scene vividly before the eyes and vests the story with new beauty. What could be better than this picture ?

> Jacob must have traversed the bleak uplands of Judæa, and still pursuing his northward way by a rough and fatiguing footpath he came at evening, just as the sun was setting, to a place, where weary and footsore, with the darkness closing in upon him, he decided to pass the night. It was a desolate spot. He had been gradually ascending, and now stood at a height of about three thousand feet above sea level. The air was keen and nipping. Around him lay a wilderness of stony fields and grey rocks, some of them piled up in weird forms of pillars, menhirs or cromlechs, while a little way off a bare hill loomed dimly skyward, its sides appearing to rise in a succession of stony terraces. He laid himself down in the centre of a circle of great stones, resting his head on one of them as a pillow, and fell asleep. As he slept he dreamed a dream.

It is not only the author's familiarity with the country of Judæa which gives these vivid touches to his descriptions, but also the fact that he is a competent Hebrew scholar. In his preface he tells us that in the preparation of his work he had read the whole of the Old Testament in Hebrew.

But in addition to these stories which Dr. Frazer is able to illuminate by an amazing fertility of cognate examples, there are many obscure references in the Old Testament which are rendered intelligible to us by analogies of beliefs and institutions culled from all parts of the world. The injunction, for example, against seething a kid in its mother's milk, which formed one of the commandments of the original Decalogue, is proved to be based upon sympathetic magic under the belief, so widespread amongst primitive pastoral folk that to apply heat to milk will dry up the udders of the herd which supplies it. Or, again, the judicial execution of an ox that had killed a man is shown to be widely practised. In the Middle Ages, law suits in due legal form were brought against flies, beetles, leeches, field mice and rats which were duly cited to appear in Court and were defended by Counsel. At Savigny, in 1457, a sow and her litter of six were indicted for the murder of a child. The parent pig was duly hanged, but as the evidence against her litter was not conclusive, their case was deferred, and their owner allowed bail for their re-appearance should further evidence be forthcoming. At Bâle, in 1474, an old cock, which had the misfortune to lay an egg, was duly sentenced to death at the stake.

Every one of Sir James Frazer's 440 pages is replete with matter of human interest both for teacher and student, priest and layman. The work is not merely an encyclopædia of human credulity; it brings together a volume of evidence that, underlying many of our cherished beliefs and practices there is a substratum of those mental processes which appear to rise universally in certain stages of primitive culture.

### FOR THE PHYSICIAN.

**A Textbook of Pharmacology and Therapeutics.** By E. Poulsson, Professor of Pharmacology in the University of Christiania. (Heinemann. 25s. net.)

This volume of five hundred pages is a translation of a well-known text-book with a high reputation on the Continent. It differs from some familiar works in the large proportion of space which is devoted to practical therapeutics. Professor Dixon, in a short preface, refers to the differences between the views held in Great Britain and on the Continent as to the value of certain remedies ; this occasional divergence of opinion has to be borne in mind, but the instances where our training and experience lead us to disagree with the author's statements are few. The volume bears the stamp of an active mind in close touch with disease in its variations with and without treatment, and for this reason is of especial value to the practising physician.

The toxic effects of the drugs described are very clearly dealt with, and measures for the avoidance or treatment of such effects are given. Thus, in speaking of chloroform administration, the view upheld by Levy that the light stage of anæsthesia is the dangerous stage is mentioned ; the harmful effect of a combination of Filix Mas and castor oil is pointed out, and the poisonous effects of arsenical and bismuth preparations are described in considerable detail. The paragraphs on these and similar questions form perhaps some of the most useful portions of the book. The reader will not find detailed accounts of the composition of all pharmacopœal remedies, and for this reason the text-book cannot be recommended as a complete guide to the student who still has to face his examiners, but it constitutes a very valuable work of reference and is packed with teaching that verily makes dry bones live. It is impossible to open the book at any page without finding some striking fact which arrests attention and compels thought. It is obvious that the author has been at considerable pains to sift the grains of gold from the dross which, while trying to usurp its value, merely debases it—his views on the commercial aspect of hormone-therapy and the value of tuberculin will find wide acceptance among thoughtful readers. A word of praise is due to the translator; she has rendered a foreign technical work into English so clear and uninvolved that it is difficult to realise that this is a translation and not the original work.

### DOES HEALTH INFLUENCE CHARACTER?

**The Relationship of Health to the Psychical and Physical Character in School Children.** By PROFESSOR KARL PEARSON. (Cambridge University Press: 15s.)

All who are interested in the education of children should read, mark, and—if they are receptive enough for unconventional and revolutionary ideas—inwardly digest Prof. Pearson's book. His investigations deal with about 4,000 boys and girls whose psychical characters are classified according as they were found by their teachers to be conscientious, keen or dull, shy or self-assertive, self-conscious or unself-conscious, noisy or quite, quick-tempered, good-natured or sullen, and popular or unpopular. Various other criteria were taken into consideration, such as handwriting, and various physical qualities. The information thus obtained is reproduced in charts and diagrams, the very sight of which will appal the average reader untrained in biometrics.

Fortunately Prof. Pearson has a facile pen and a capacity for conveying in a few words simple and salient facts. He has come to the conclusion that his findings shatter three widely held beliefs. Firstly, great intelligence and a variety of psychical characters are the gift of the parents and not of the teachers; the parents provide the metal, and all the teacher can do is to give it an edge and temper it. Secondly, general health changes little during the whole school period, and it does not seem feasible with the present state of medical knowledge to improve intelligence by modifying health. Thirdly, there appears to be no foundation for the widespread opinion that health is a governing factor of temperament. Enthusiastic teachers may claim that education, and sanguine medical men may claim that improved health, modify the psychical characters of children, but cold statistical light does not support them.

"Is it not blind audacity," Prof. Pearson asks, "for man to claim that by aid of his puny, if undoubtedly useful, little polishing tools, such as education and medicine, he can achieve as much in a school life as Nature with her omnipotent mechanism has taken in all probability a quarter of a million years to produce?" If Prof. Pearson is not very careful, he will soon be known as The Gloomy Doctor.

### MYSTIC MALGRÉ LUI.

**Health and the Human Spirit.** By K. W. MONSARRAT, F.R.C.S.ED. (Murray: 5s.)

This "biological study" is a plea for the employment of the scientific method in the worlds of thought and affairs no less than in the world of medicine. It argues that there is no other means of knowledge or road to certainty; that philosophy and "revealed" religion, in so far as they do not employ it, continue to mistake desires for facts and are doomed to disappointment. There is a quality of restrained ardour in these pages that shows the author to have a genuine insight into the meaning of the word "science," and he is one of those always interesting and not too common examples of a mind in which the inductive and the poetic faculties do not exclude each other.

It is odd, therefore, that he has failed to realise that this conjunction is precisely that which has been the quality of the great mystics from St. Catherine of Siena to Florence Nightingale. Both had, in their different ways, immense visionary power; both were great administrators and statesmen. Yet Mr. Monsarrat allows no mercy to the mystics, though his point of view, especially in his concluding chapter, is one that is unmistakably akin to theirs. He is always inviting us to attend solely to reality, and a mystic has been defined as one who has had a first hand apprehension of it, as Newton had of scientific law. Mr. Monsarrat has written verses; he claims the artist and the savant to be equally men of science. Surely, therefore, he knows that, taking words in their ordinary meaning, "intuition," on which he is severe, is a better term for the artistic perception of truth than the "intelligence" which he prefers to it. We mention this because the defect of the book is an undue simplification. If we apply the scientific method that he rightly exalts to any problem of the hour—to divorce, say or the capital levy, or the prevention of venereal disease—we find it inadequate to a solution, not because the method is wrong, but because the facts are obscure, or confused or multitudinous.

The attitude which he commends is right, but it does not work so simply as his essay would imply, except in comparatively restricted fields of investigation. Yet the man who has been trained in medicine will sympathise with a surgeon's attempt to apply the scientific method to matters social, political or philosophic, from which it is too often debarred.

### A NURSE'S LETTERS FROM RUSSIA.

**Plague, Pestilence and Famine.** By MURIEL A. PAYNE. (Nisbet. 3s. 6d. net.)

In offering her services to the Society of Friends for relief work in Russia, Miss Payne described herself as "a trained nurse, qualified health visitor, organiser of clinics, a bad typist, a shocking speller, a driver of Ford lorries, used to roughing it, strong as a horse, late supervising sister of Child Welfare work in Czecho-Slovakia." Spelling and typewriting are clearly of little importance in the devastation of Russia, but the amazing energy, steady cheerfulness and robust common-sense of which Miss Payne is obviously possessed must have been of untold value to the starving and forsaken people among whom she worked. This book is a collection of letters written in the midst of strenuous labours and unceasing worries, and are wonderfully impressive in their stark simplicity. From Moscow in March of last year she writes:—

> I am at the present moment acting as milkman, and delivering milk for 15,000 children at the institution in Moscow, also cases of soup and cooking utensils. Wood is so expensive and difficult to get that there is very little hot water, and no soap can be bought. The condition of the hospitals is quite indescribable. There are often three patients in each bed.

and sick people laying all up the middle of the wards. The most terrible place is the Epileptic Hospital, where the children are all starving because the Relief Organisations and the Government feel that the food is so precious that it should be kept for the children worth saving. . . . This morning I went to see the refugees in Moscow station. There were hundreds of people lying or sitting on their luggage, huddled on the top of each other for warmth, or picking the lice off each other. In one room the sick people were collected —most of them suffering from typhus or recurrent fever. One doctor struggles daily to try and find room for them in the hospital, but many die just where they are.

Miss Payne's district consisted of six counties with a population of 67,000. The sanitary arrangements were appalling—the water thick with black weeds from the river in which there were floating bodies. The children, even when they had food, died by the dozen of typhus, dysentery and pneumonia. There was no clean linen to put on their sores, and they lay on bare boards with only rags to cover them :—

Last Sunday we went for a walk by the river. We took a little food with us, and as we sat and ate it I suddenly saw the hand of a dead child lying on the bank. Another day we passed two men in a disused house, half eaten. We notified the Health Authorities, but four days later they were still unburied, and seemingly there is no chloride of lime in the country.

In one hospital were living one doctor, four unqualified assistants, twenty-seven nurses, their families, and twelve patients, all more or less starving. As they had no food they could not take in more patients or do any work. Nothing was left except bare iron beds with wooden planks on them. The surgical cases were all septic, and there were no dressings. This " hospital " Miss Payne set herself to reorganise :—

I promised rations if they would clean up, mend beds and generally rearrange things. In a week we had achieved a fairly decent hospital of seventy beds, though there are no sheets, blankets, dressings or drugs ; but the wards are warm and the Communists have come to the fore splendidly, obtaining wood and arranging for electric light and the water supply. We are all bursting with pride over our sheetless hospital and have planned it out as follows : General block with large children's ward and maternity ward ; typhus block with twenty beds ; cholera block with six beds. The mattresses, sheets, towels and nurses' uniforms are all made of sacks ; the nurses are all absolutely untrained ; but it is a haven, anyhow, for a few people to come to, and will, at any rate, isolate the sick.

Miss Payne says truly that a " nurse's work is essentially unpolitical, undenominational and international," and then proceeds unmercifully to criticize the Russian Church ! Generally speaking, however, she is fair-minded enough, and the book certainly gives one of the most vivid and terrible accounts of Russia that we have yet read.

### THE ASTHMATIC.

**Asthma.** By Frank Coke, F.R.C.S. (John Wright & Sons, Ltd., Bristol ; 15s. net.)

The paroxysmal phenomena associated with what is commonly known as spasmodic asthma have always excited the interest of physicians and physiologists. Of late years, however, our knowledge of the ætiology of asthma has been considerably enriched by experimental researches in this country and America. In the present volume the author not only reviews the various theories and experiments which may be regarded as more or less explanatory of asthma as a symptom, but has recorded the results of treatment of upwards of three hundred cases examined and treated in the light of this new work. He rightly pours contempt upon the wholesale use of the expression " nervous manifestation " or " temperament," in connection with the asthmatic and his malady. So numerous are the influences at work in different individuals that it is necessary to take a most careful history in each case to determine what is the nature of the patient's special sensitiveness. The discovery of the sensitisation to foreign proteins of many asthmatics has opened the door to a new form of special diagnosis and therapy, of which a succinct account is here given by Mr. Coke.

In his series of dermal tests, 52 per cent. gave positive reactions, and it may be noted that the smallest reaction allowed was an unmistakable plus one. Thus, in one case to which he refers, a child gave a " five-plus " reaction to cat hair, and a " plus-minus " reaction to potatoes, but it was the cutting off the potatoes that finally cured him. The word " adzyme " is coined by the author to denote that factor in the blood or tissues that is responsible for the process whereby the subject becomes sensitive to a foreign protein. The mechanism of the dermal reaction and the manner of applying the special tests are fully described, as well as the methods of cure by specific protein therapy, or desensitisation by small doses, and by vaccine and non-specific protein therapy. The author concludes that no case of asthma need be despaired of, provided its cause be investigated thoroughly. We heartily recommend this monograph to the notice of all medical practitioners and senior students.

### " KIRKES " UP-TO-DATE.

**Handbook of Physiology.** By Prof. W. D. Halliburton, F.R.S. (Murray. New edition. 21s. net.)

When, in 1842, it was decided at St. Bartholomew's Hospital to separate the teaching of the subject of physiology, then in its early infancy, from that of anatomy, of all the staff James Paget alone had the requisite knowledge. He was accordingly elected unopposed to the first lectureship in physiology in the medical school. Of English text books there were none, and in 1848 one of his own pupils, Dr. William Kirkes, supplied this need, drawing his material very largely from the lectures and notes of Paget.

Seventy-five years have elapsed and still *Kirkes' Physiology*, though now under another name, holds its place. The book has, in the interval, passed through twenty-eight editions, for the last sixteen of which Prof. Halliburton has been responsible. Generations of students have pored over its pages, and the title, *Kirkes' Physiology*, will doubtless conjure back to hundreds of middle-aged practitioners memories of happy days of callow youth and all the camaraderie of student life. The indefinable quality which has conferred such vitality upon a book of this kind is not easy to put into words, though it clearly depends upon the capacity of the editor to keep abreast of the recent advances in the subject and to explain them in their proper relation to the whole science. The book has none of those finely-finished rolling sentences which used to make the reading of Prof. Michael Foster's *Physiology* such a delight. On the other hand the whole subject matter is so set forth that the interest of the reader is always aroused and sustained—a quality which is further aided by the abundance and excellence of the illustrations, of which there are nearly six hundred. The historic element is also maintained in the present edition by the inclusion of portraits of famous physiologists.

Of the subject matter we need say little. The whole book has been recast with but little increase in actual size and the latest physiological developments have been incorporated—for example, the discovery of insulin and the protopathic and epicritic theory of sensibility of Head and Revers. The section on reproduction has had the advantage of revision by Dr. J. H. Marshall.

### NURSING TUBERCULOUS CHILDREN.

**The Nursing of Surgical Tuberculosis in Children.** By Janet P. Robertson. With Foreword by Sir Henry Gauvain, M.A., M.D., M.C. (The Scientific Press, Ltd., London. 3s. net.)

This clearly written and profusely illustrated little volume is largely a reprint of lectures that appeared originally in The Nursing Mirror, and forms an excellent yet simple exposition of the treatment, both general and local, of surgical tuberculosis, as carried out at the Lord Mayor Treloar Cripples' Hospital, Alton. The author, who has been for fifteen years Matron of the hospital, has had a unique experience in dealing with children suffering from that most common variety of the disease, tuberculosis of the bones and joints, and to her intelligent co-operation and clever management Sir Henry Gauvain, the Medical Superintendent of the hospital, pays a warm tribute in his preface.

The chapters on plaster-work will be most helpful to the learner entering this interesting branch of her profession, and also to those already in charge of an institution run on the same lines as that at Alton, while to anyone seeking to establish a similar centre of work, the final chapter on "Administration" will strongly appeal. It is so evidently the outcome of Miss Robertson's own ripe experience, and so full of wise hints and sound advice, that it should be invaluable to others who may be only beginning to tread the somewhat thorny path of the hospital administrator.

### MISCELLANEOUS NOTICES.

### A Director of Midwives.

Practising Midwives and Maternity Nurses. (The Scientific Press, Ltd., 9d. net.)—This little directory has been compiled in response to repeated requests and to facilitate easy reference is divided into three sections—Certified Midwives Practising Alone, Certified Midwives Practising with Doctors and Maternity Nurses. It is thus most easy of access and should be distinctly useful.

### Lymphadenoma.

Hodgkin's Disease. By R. Allan Bennett, M.D. (John Wright and Sons, Ltd., Bristol. 1923. 2s. net.)—In this little monograph the author seeks to define what is strictly meant by Hodgkin's disease, and while following the description given by Andrewes he lays stress upon the changes in the stroma of the lymphatic glands as being characteristic of this affection. In spite of the conflicting evidence as to the possible tuberculous origin of Hodgkin's disease, Dr. Bennett is inclined to give it a place by itself. In his hands X-rays and creosote inunction have proved of some service.

### For Mothers.

The Vigil of Hope. By F. A. Macleod. (Longmans. 3s. 6d. net.)—Miss Macleod, while Librarian to the Mothers Union, received constant requests for a book of prayer and meditations for mothers awaiting the birth of their baby, and in this little volume she has endeavoured to meet an evident need. These brief and practical meditations and simple litanies should certainly supply just what is required.

### Biographical Sketches.

English Physicians of the Past. By R. T. Williamson, M.D., F.R.C.P.. (Reid, Newcastle).—These short sketches of the life and work of twelve famous physicians are written for "junior medical men and students who have little time or opportunity for the study of medical biography." They are quite admirable, within their necessarily restricted limits, and should certainly arouse in their readers a desire for further knowledge of the lives and characters of such men as Linacre, Harvey and Sydenham.

### The Problem of Ventilation.

The Ventilation of Public Buildings. By Robert Boyle. (Boyle and Son. 6s. net.)—This attractively bound and well illustrated little book consists mainly of extracts from various authorities on the merits of natural ventilation. These authorities include such famous scientists as Lord Rayleigh, Lord Kelvin, Lord Lister and Sir William Crookes. Yet, writes Mr. Boyle in his Preface: "That method also has its limitations; indeed, it would appear to be more than doubtful if any form of ventilation will ever be devised that will achieve perfect ventilation under all conditions or that will satisfy all idiosyncrasies." It certainly seems too much to hope for. The costliest system of ventilation in use in this country is that of the House of Commons, and it is incomparably the worst. It gives honourable members cold feet and hot heads, which is the exact opposite of the real needs of legislators.

### A Text-Book of Otology.

A Practical Handbook of Diseases of the Ear. By William Milligan, M.D., and Wyatt Wingrave, M.D. (William Heinemann. 1923. 12s. 6d. net.)—This is essentially a book for students, and as such is sufficiently dogmatic in its teaching to give a clear indication as to the diagnosis and treatment of each morbid condition described. The methods of examination and the instruments employed for the investigation of the causes of deafness are given in considerable detail. Affections of the external auditory meatus and mastoid disease are described at some length, whilst the last chapter contains numerous useful formulæ and hints for the performance of certain laboratory tests. Altogether this book may be recommended as a sound guide to the principles and practice of otology.

### Vicious Circles in Pathology.

Les Cercles Vicieux en Pathologie. Traduit sur la troisième édition Anglaise. Par Dr. C. Flandin et Dr. F. Françon. (Paris: A. Maloine et Fils.)—This is a translation from the third edition of the English work of Dr. J. B. Hurry, of Reading, on vicious circles in disease. The translation having been supervised by Dr. Hurry, it may fairly be claimed that this is a fourth edition of his notable work. The title alone epitomises the contents of this book. It has long been taught that certain morbid conditions not only act, but react, on each other. It has, however, been reserved for Dr. Hurry to systematise this idea, and in his well-known English editions he has elaborated his conception of vicious circles in disease in a manner both scientific and scholarly. Most books on medicine have, or attempt to have, a scientific tone. But it is very rare at the present time to find medical books showing in every page that touch which only the scholar can give. Scientific works are not translated into foreign languages on account of the author's purely literary qualities, and this French translation is an eloquent tribute to the intrinsic value of the ideas of which Dr. Hurry is so able a spokesman.

Messrs. Allen and Hanbury publish a third revision of their hardy little pamphlet on "Infant Feeding in the Light of Recent Research."

